# A weekly‐diary study of students' schoolwork motivation and parental support

**DOI:** 10.1111/bjep.12532

**Published:** 2022-07-31

**Authors:** Yao Wu, Peter Hilpert, Harriet Tenenbaum, Terry Ng‐Knight

**Affiliations:** ^1^ School of Psychology University of Surrey Surrey UK

**Keywords:** parental support, schoolwork motivation, weekly diary study

## Abstract

**Background:**

Parental support plays an important role in children's schoolwork motivation and may have been even more important during the first UK COVID‐19 pandemic lockdown because all schoolwork was completed at home. When examining the effect of parental support on children's schoolwork motivation, research has typically focused on comparing families with each other (i.e., difference between families). In reality, however, the effect unfolds as a transactional, bidirectional process between parents and children over time (i.e., a within family process). This research trend can result in imprecise conclusions about the association between parental support and schoolwork motivation.

**Objectives:**

We examined bidirectional effects of parental schoolwork support and children's schoolwork motivation at both the between‐family and within‐family level.

**Methods:**

This study reports findings from a weekly‐diary study conducted during the first UK COVID‐19 school lockdown. Cross‐lagged within and between multilevel modelling was used to analyse data from UK secondary school students (*N* = 98) in Years 7–9.

**Results:**

Between‐family results show no evidence of association between motivation and parental support. Within‐family results indicate that higher motivation (assessed as higher expectations of success) predicted more support from parents. However, in contrast with predictions, weekly levels of parental support did not predict children's weekly fluctuations in motivation.

**Conclusions:**

Within‐family results were not consistent with between‐family results. This study is novel in showing that child‐driven effects appear to be important in eliciting parental support within families over time.

## BACKGROUND

Schoolwork motivation is an important predictor of academic achievement (Trautwein & Lüdtke, 2007). Parental support of children's schoolwork plays an important role in promoting students' academic motivation (Moroni et al., 2015; Silinskas & Kikas, 2019a; Xu et al., 2018). Families differ in how much support parents provide. Families offering more support tend to have more motivated children. However, the dynamic interplay of how parental support and children's schoolwork motivation influence each other bi‐directionally, within families over time, are not fully understood (Farooq & Asim, 2020; Hoglund et al., 2015). Furthermore, the association between support and motivation may have been amplified during the COVID‐19 period because parents spent more time with their children during school closures. By improving understanding of within‐family mechanisms underpinning parental support and schoolwork motivation, we may be able to develop more effective interventions. This study explores variations in schoolwork motivation on a weekly basis to gain a better understanding of the relationship between parental support and schoolwork motivation during the first COVID‐19 school lockdown in England.

## Schoolwork motivation

Schoolwork motivation plays a critical role in academic achievement (Dinkelmann & Buff, 2016; Feng et al., 2019; Lazarides et al., 2019). According to expectancy‐value theory (EVT; Eccles, 1983), schoolwork motivation is composed of two components ‐ expectancy and value beliefs. Expectancies refer to how well students believe they can successfully perform achievement‐related tasks (Eccles & Wigfield, 2002, 2020), whereas students' value beliefs measure the extent to which individuals think an academic task is worthwhile and valuable (Eccles, 1983; Eccles & Wigfield, 2020).

Existing research recognizes that individual differences in schoolwork motivation predict academic achievement (Hattie, 2009; Murayama et al., 2013). However, this work is mostly restricted to examining motivation differences between students. Recent explanations of expectancy‐value theory (i.e., situated expectancy‐value theory, SEVT; Eccles & Wigfield, 2020), highlight that expectancy and value beliefs not only differ across individuals (between‐person level) but also within individuals over time (within‐person level; Wigfield et al., 2015). Like state–trait perspectives, SEVT proposes that motivation consists of a trait‐like component that does not easily change over time, and a state‐like component where expectancy and value beliefs vary within an individual over time and across situations (Eccles & Wigfield, 2020). For example, a between‐person level trait is when a child has a higher expectancy for English compared to other children on average, whereas a within‐person level state is when a child has lower English schoolwork expectancy in a given time point than they themselves typically do. Currently, little is known about the causes of within‐person fluctuations in motivation over time. To address this gap, the present study examined how motivation fluctuated within person over time during the UK's first COVID‐19 school lockdown and tested the role of perceived parental support as a predictor of weekly fluctuations in motivation.

## Parent‐to‐child effects

Parental support in children's schoolwork is related to schoolwork motivation (Froiland, 2015; Ma et al., 2016; Puklek Levpušček & Zupančič, 2009). Parental support can be described as providing support when requested and being available to help solve problems with schoolwork (e.g., giving hints and ideas; Feng et al., 2019; Pomerantz et al., 2007). Self‐determination theory also informs the current study to conceptualize processes relating to parental support in children's schoolwork behaviour (Doctoroff & Arnold, 2017). This theory posits that parental behaviour that satisfies children's basic psychological need for autonomy (freedom to feel, think, or act), competence (feelings of effectiveness and mastery), and relatedness (feeling of connection with others) contributes to facilitating children's academic motivation and skills (Ryan & Deci, 2000; Silinskas & Kikas, 2019b; Xu et al., 2018). Parental support can benefit students' academic motivation because a sense of autonomy and competence might increase in children when their parents provide children opportunities to make choices and encourage active problem solving.

Parental support has been found to positively affect children's academic motivation (Vasquez et al., 2016; Xu et al., 2018). Intervention research has shown that improvements in parental support increased children's homework motivation in 4th to 5th grade children (Froiland, 2011). Longitudinal research over a two‐year time frame tracking parent‐teacher‐child triads linked higher levels of parental support to higher academic motivation (Viljaranta et al., 2018). Most studies in parental schoolwork involvement are either cross‐sectional or longitudinal with long‐time intervals (often spanning multiple years; Dumont et al., 2014; Xu et al., 2018). Such approaches, however, have failed to look at dynamic parental support, that is, changes and fluctuations in support from week to week, and cannot separate between‐person and within‐person processes. Current theoretical models imply that the associations between children's schoolwork motivation and parental support occur both between persons and within persons (Skinner et al., 2019). Research has indicated the importance of examining within‐person variation that stresses covariation within a person over time (Dietrich et al., 2017; Eccles & Wigfield, 2020). In addition, capturing different reactions in the same individual in different situations is central to psychological processes. Failing to make this distinction may result in an imprecise understanding of the relation between motivation and parental support. More specifically, a child's motivation may depend more on whether their parent provides more support for schoolwork during a particular week when support is needed than if a parent's average level of support differs from other parents' level of support. In the present study, weekly diary measures from the same participants provided a unique perspective on dynamic parental schoolwork support processes over time. Following research on motivation (Dumont et al., 2014; Karbach et al., 2013; Núñez et al., 2017), we included children's perceptions of parental support because children's perceptions are central to their motivation.

## Child‐to‐parent effects

Beyond the unidirectional influence of parents on children's academic motivation, many developmental theories argue for inclusion of bidirectional or transactional processes between parents and children (Sameroff & Mackenzie, 2003; Skinner et al., 2019). Transactional processes are continuous dynamic reciprocal exchanges involving children, experiences provided by their parents, and the social context. These exchanges produce a series of bidirectional effects on both children and the context (e.g., parents; Sameroff, 1975). Longitudinal research supports the existence of transactional processes, for example, low academic achievement predicts increased parental assistance over time (Hoglund et al., 2015; Núñez et al., 2017; Silinskas et al., 2013). Using a cross‐lagged model between children's academic achievement and parents' academic expectations during primary school, Briley et al. (2014) found bidirectional effects. These findings are consistent with the transactional framework in which parent‐to‐child effects and child‐to‐parent effects co‐occur. Children's motivation and academic achievement may shape parental schoolwork support, which in turn, predicts children's subsequent academic motivation and performance.

Research on bidirectional and transactional effects is crucial to illuminate parent–child interaction. However, most previous studies have only focused on parents' effects on schoolwork motivation and failed to investigate the transactional nature of children's schoolwork motivation and parental support unfolding over time (Vasquez et al., 2016; Xu et al., 2018). Failing to capture bidirectional relations may result in an imprecise understanding of the association between parental support and motivation. Additionally, research must distinguish between‐person and within‐person processes. Between‐person processes are easily misinterpreted as within‐person processes, leading to mistakenly generalizing between‐person differences to within‐person results (Hamaker et al., 2018; Voelkle et al., 2014). Moreover, understanding the pattern and causes of within‐person changes may help to identify more appropriate intervention targets. The current study tests how children's schoolwork motivation both responds to and triggers parental schoolwork support at the between‐person and within‐person levels. Thus, we considered the following research question: how do children and parents influence each other within families over time and across families?

## The present study

The link between parental support and schoolwork motivation may have intensified during the lockdown because school closures forced parents to take responsibility of their children's schooling (Andrew et al., 2020; Bubb & Jones, 2020; Green, 2020). Moreover, the increased time parents spent with their children on schoolwork made this an ideal time to assess how parents and children affect each other during schoolwork. In addition, children's schoolwork motivation might have fluctuated during the lockdown due to less physical contact with teachers and peers as well as more passive screen activities (Bayrakdar & Guveli, 2020). During the UK COVID‐19 lockdown in Spring 2020, pupils in the age range in the present study completed all their schoolwork in the home context. Hence, in this study, we use the term, ‘schoolwork’ rather than ‘homework’.

To examine how children's schoolwork motivation varies during a period of school closures and advance our understanding of the bidirectional effects of parents and children at between‐person and within‐person levels, we used a weekly diary design to measure children's schoolwork motivation and parental support in English and maths during five consecutive weeks. Data were analysed with double‐intercept multilevel models to identify the between‐person (trait) and within‐person (state) processes, thereby shedding light on the complex interplay of schoolwork motivation and parental support. We proposed the following hypotheses:

## Between‐person hypotheses

Based on research focused at the between‐person level such as Heatly and Votruba‐Drzal (2017), Silinskas and Kikas (2019b), and Wei et al. (2019), we expected that children who perceive higher than average levels of parental schoolwork support would have higher than average levels of schoolwork motivation (assessed as both expectancy beliefs and value) in comparison to children who perceive lower than average levels of parental schoolwork support. We also hypothesized that individuals who report higher than average levels of schoolwork motivation, tend to receive higher than average levels of parental schoolwork support in comparison to students who report lower than average levels of schoolwork motivation.

## Within‐person hypotheses

At the within‐person level, we hypothesized that when children received higher parental support in one week, they would report higher schoolwork motivation in the subsequent week in comparison to a week where they received lower parental support. We also expected that individuals with higher schoolwork motivation in one week would receive more parental support in the subsequent week.

## METHOD

### Participants

Participants included 98 secondary school children from Years 7 to 9 (52 girls, 46 boys) and one of their parents. All participants were recruited using email through schools or online advertisements. For the children's questionnaire, responses were obtained from 48 children at week 1 (51% missing), 72 children at week 2 (24% missing), 78 children at week 3 (20% missing), 76 children at week 4 (22% missing), and 65 children at week 5 (34% missing). Most parental responses were from mothers (90.59%). There were 28 participants who had five data points, 33 participants who had four data points, nine participants who had three data points, 11 participants who had two data points, five participants who had one data point, and 12 who did not answer the schoolwork motivation questions. Students' ages ranged from 11–15 years (*M*_*age*_ = 12.70 years, *SD* = 1.04 years). Among the students, 47% were in Year 7 (first year of secondary school), 22% were in Year 8, 29% were in Year 9. Most children in the sample were of white ethnicity (see Table 1). Most children did not have (57 of 85 responses) or attend (61 out of 85 responses) synchronous online English and Maths lessons.

**TABLE 1:** Demographic information

	*N*	%
Gender		
Girl	52	53.1
Boy	46	46.9
Other	0	0
Age		
11	9	9.2
12	41	41.8
13	21	21.4
14	24	24.5
15	3	3.1
School year		
7	47	48.0
8	22	22.4
9	29	29.6
Eligible for free school meals		
Yes	5	5.1
No	71	72.4
Missing	22	22.4
Ethnicity		
White	59	60.2
Mixed/multiple ethnic groups	8	8.2
Asian/Asian British	5	5.1
Black/African/Caribbean/Black British	0	0
Other	3	3.1
Missing	23	23.5
School type (I)		
Private	6	6.1
State	79	80.6
Missing	13	13.3
School type (ii)		
Comprehensive (non‐selective)	64	65.3
Grammar (academically selective)	19	19.4
Missing	15	15.3

### Procedure

This weekly diary study was conducted during the first UK COVID‐19 lockdown. Schools were physically closed to students in this age range with an exception for children of key workers and children with special educational needs.

We used two methods of recruitment. First, we emailed schools asking them to send out an advertisement about our study to parents in their newsletters. Six schools agreed to include our information in school newsletters. The six schools were in southeast England and are broadly representative of English secondary schools. Second, we placed advertisements about the study on social media (Twitter) and a website (Childrenhelpingscience.org) set up during the pandemic to recruit participants for studies. We included the researcher's email address and sign‐up link in our information. The sign‐up form asked the parent to provide email addresses so we could send a link to the weekly questionnaires. Data were collected through online questionnaires via Qualtrics. Data collection started on 19th June 2020 (week 13 of the UK lockdown) and was completed on 20th July 2020 (the final week before school summer holidays; week 17 of the UK lockdown). During five consecutive weeks, students and parents received an invitation email including the questionnaire link at 10 am every Friday. The weekly questionnaires covered the children's weekly schoolwork experience during the COVID‐19 pandemic, etc. In addition, demographic questions were included at the start of the study (the first questionnaire that participants completed). Therefore, the total duration for the children's weekly questionnaire was around 20 min for the first time and 15 min for all subsequent weeks. For the parents' weekly questionnaire, the total duration was about 15 min for the first questionnaire and 10 min for all subsequent weeks. To increase the responses to the questionnaires, families who completed half the weekly questionnaires received £10 in vouchers, and families who completed all weekly questionnaires received £20 in vouchers. In the current study, we only include demographic information from parents.

Students and parents could complete the questionnaire between Friday at 10 am and the following Monday at midnight. To improve completion rates, reminder emails were sent to participants every Saturday and Monday at 10:00 am. Most parents completed the questionnaires on Fridays, and most children completed questionnaires on Saturdays. Table 2 displays the number of valid responses for all time points.

**TABLE 2:** Participants' involvement across five weeks

	Distributed questionnaire	Completed questionnaire *N*	Completed questionnaire %
Week 1			
Child	61	48	79%
Parent	61	46	75%
Week 2			
Child	99	74	75%
Parent	99	81	81%
Week 3			
Child	103	78	76%
Parent	103	85	83%
Week 4			
Child	100	76	76%
Parent	100	85	85%
Week 5			
Child	95	65	68%
Parent	95	76	80%

### Measures

#### Schoolwork motivation

Students' schoolwork motivation was measured by self‐report every week and was formed of two subscales assessing expectancy and value beliefs. Expectancy and value beliefs were assessed for two core school subjects, mathematics and English. Expectancy beliefs were assessed with eight items adapted from a measure used in Trautwein et al. (2006). Four items of expectancy were reversed scored. Scores for both maths and English showed high internal reliability (mean Cronbach's alpha across five weeks, α_English_ = .86, α_Maths_ = .89).

To measure the value component, we adopted eight items from the Task Value Scale (Gaspard et al., 2017) which included items assessing the facets of intrinsic value, attainment value, utility value, and cost (cost items were reversed scored because high cost indicates low value). Mean scores for value showed high internal reliability for both maths and English (mean Cronbach's alpha across 5 study weeks: α_English_ = .82, α_Maths_ = .83). All items utilized a 4‐point Likert scale from 1 (*Completely disagree*) to 4 (*Completely agree*). High scores on these measures indicate high expectancy beliefs and high value beliefs. The schoolwork motivation (expectancy and value beliefs) items are reported in the Table 3.

**TABLE 3:** Items assessing schoolwork motivation and parental schoolwork support

Schoolwork motivation
Expectancy beliefs
If I made an effort, I could do all my [subject] schoolwork
2 I often felt completely lost in my [subject] schoolwork. (R)
3 If I had difficultly [subject] doing my schoolwork, I knew where to look to find the right answer
4 When I was trying to do my [subject] schoolwork, I often thought I would never understand it. (R)
5 If I did not understand something in [subject] schoolwork, I was at a complete loss and did not know how to catch up. (R)
6 I sometimes really feared my [subject] schoolwork. (R)
7 If I wanted to, I would always find a way to do my [subject] schoolwork correctly
8 If I did not understand something in my [subject] schoolwork, I knew where to look it up
Value beliefs
I enjoy my [subject] schoolwork
2 My [subject] schoolwork is fun
3 Doing my [subject] schoolwork is important to me
4 I care about my [subject] schoolwork
5 Good grades in [subject] can help me in the future
6 Learning [subject] is worthwhile, because it improves my job and career chances
7 Doing my [subject] schoolwork makes me really tired. (R)
8 Doing my [subject] schoolwork stops me having fun. (R)
Parental schoolwork support
My parents helped me with my [subject] schoolwork if I asked them to
2 My parents helped me with my [subject] schoolwork if I was having difficulties

*Note*: All items were scored from 1 (Completely disagree) to 4 (Completely agree). *R* = item was reverse scored.

#### Parental schoolwork support

Two items were adapted from Dumont et al. (2014) to assess children's perceptions of parental schoolwork support weekly (α_English_ = .92, α_Maths_ = .95). In weekly studies, where participants have to complete the same questionnaire multiple times, it is common practice to use fewer items (Hilpert et al., 2018). Although the scale may be less reliable because of fewer items, the reliability is at the same time increased because the same participants complete the same items at multiple times. Participants responded using a 4‐point Likert scale ranging from 1 (*Completely disagree*) to 4 (*Completely agree*). High scores on these items indicate more parental schoolwork support. Table 3 lists the items.

#### Demographics

Children's race (coded 1 = White, 2 = Mixed/Multiple ethnic groups, 3 = Asian/Asian British, 4 = Black/African/Caribbean/Black British, 5 = Other), free school meal eligibility, and school types were reported by parents when they completed the first questionnaire.

### Data analysis strategy

The main aim was to disentangle the reciprocal relationship between children's schoolwork motivation (expectancy and value) and their perceived parental schoolwork support at the between‐person and within‐person level. Cross‐lagged between‐ and within‐level models were used to analyse the nested data (Bolger & Laurenceau, 2013; Hamaker et al., 2015; Raudenbush & Bryk, 2002). In the first model, children's expectancies about how well they would perform in English and their perceived parental schoolwork support were separated into between‐person and within‐person components. To compute the between‐person component, a mean was computed for each participant over the five weeks (Expectancy_between_, Support_between_). To obtain the within‐person component, a person's mean was subtracted from the individual's value for each week (Expectancy_within_t_, Support_within_t_), representing weekly variations from a person's mean. Furthermore, we computed a lag variable for each within‐person component (e.g., Expectancy_within t+1_). This variable allows us to predict how expectancy and support in week t (Expectancy_within t_, Support_within t_) predicts expectancy in the subsequent week t + 1 (Expectancy_within t+1_). Thus, this approach allows us to compute auto‐regressive effects (expectancy in week t affects expectancy in the following week t + 1) as well as cross‐lag effects (expectancy in week t affects support in the subsequent week t + 1).

We used a double intercept model (Bolger & Laurenceau, 2013), because this model allows us to compute the effects for the two dependent variables simultaneously (Expectancy_within_t+1_, Support_within_t+1_). This strategy requires an analytical strategy involving dummy codes (0/1) and the use of interaction terms with the dummy codes to compute the effects for both dependent variables Expectancy_within_t+1_ or with Support_within_t+1_ (for details see Bolger & Laurenceau, 2013). The data of the predictors are doubled via the dummy code that defines whether the coefficients for Expectancy_within_t+1_ or with Support_within_t+1_ are computed. The equation for the multilevel model is shown below.

$$\begin{array}{c} Y_{\text{within}\_\mathrm{it}+1}={{\left(\text{dummy}=0\right)}_{i}}^{*}[\gamma_{00}\left(\text{time}\right)\\ \;\;+\gamma_{01}\left({\text{Expectancy}}_{\text{between}}\right)+\gamma_{10}\left({\text{Expectancy}}_{\text{within}\_t}\right)\\ \;+\gamma_{02}\left({\text{Support}}_{\text{between}}\right)+\gamma_{20}\left({\text{Support}}_{\text{within}\_t}\right)\\ \;+u_{p0i}+u_{p1i}\left({\text{Expectancy}}_{\text{within}\_t}\right)+u_{p2i}\left({\text{Support}}_{\text{within}\_t}\right]\\ \;+{{\left(\text{dummy}=1\right)}_{i}}^{*}[\gamma_{03}\left(\text{time}\right)\\ \;+\gamma_{04}\left({\text{Expectancy}}_{\text{between}}\right)+\gamma_{30}\left({\text{Expectancy}}_{\text{within}\_t}\right)\\ \;+\gamma_{05}\left({\text{Support}}_{\text{between}}\right)+\gamma_{30}\left({\text{Support}}_{\text{within}\_t}\right)\\ \;+u_{c0i}+u_{c1i}\left({\text{Expectancy}}_{\text{within}\_t}\right)+u_{c2i}\left({\text{Support}}_{\text{within}\_t}\right]+\varepsilon_{\mathrm{it}} \end{array}$$

In this double intercept multilevel model, Y represents the dependent variables (Expectancy_within_t+1_, Support_within_t+1_), i indexes individual, and t indexes week. γ_00 and_ γ_03_ indicate the two fixed intercepts. γ_01,_ γ_02,_ γ_04,_ and γ_05_ represent between‐person level fixed effect. γ_10_, γ_20_, γ_30_ and γ_30_ represent the within‐person level fixed effect. u_p0i_ and u_c0i_ indicate person‐specific random intercepts. u_p1i,_ u_p2i_, u_c1i_ and u_c2i_ represent the random slopes of Expectancy_within_t_ and Support_within_t_ respectively. ε_it_ stands for the regression residual for person and week.

Statistically, the same model was used for three additional models. Model 2 included the same constructs but with children reporting their expectancy and support towards Maths. Model 3 focused on the constructs of value and support about English, whereas Model 4 focused on the constructs of value and support about Maths.

SPSS version 26.0 was used for descriptive analyses. Multilevel cross‐lagged model analyses were performed in R software version 3.6.3 (R Core Team, 2019) using the tidyverse (Wickham et al., 2019), lme4 (Bates et al., 2014), and parameters (Lüdecke et al., 2020) packages. Although multilevel modelling cross‐lagged modelling was deemed the most appropriate approach to data analysis, for completeness, alternative analytic approaches were explored. Missing data are common in longitudinal studies because individuals may be absent at single time points or there may be nonresponses to single items. R can handle missing data via Full Information Maximum Likelihood. This analysis is superior to analyses that use listwise deletion methods (Schafer & Graham, 2002).

## RESULTS

### Preliminary analyses

Overall, levels of schoolwork motivation (Expectancy and Value) and parental schoolwork support were high. Table 4 displays the means, standard deviations, and correlations for key variables.

**TABLE 4:** Descriptive statistics

Variable	*M*	*SD*	Expectancy (English)	Value (English)	Parental support (English)	Expectancy (Maths)	Value (Maths)	Parental support (Maths)
Expectancy (English)	3.34	0.59	–	.33*	.14*	.46*	.18*	.14*
Value (English)	3.05	0.58	.61*	–	.14*	.24*	.40*	.14*
Parental Support (English)	3.51	0.75	.11*	.17*	–	.08	.17*	.52*
Expectancy (Maths)	3.35	0.62	.71*	.40*	.12*	–	.50*	.14*
Value (Maths)	3.08	0.58	.51*	.72*	.16*	.69*	–	.22*
Parental Support (Maths)	3.53	0.79	.07	.04	.66*	.21*	.08	–

*Note*: Means, SDs averaged across five weeks of the study. *p < .05. Correlations at the within‐level are above the diagonal, and correlations at the between‐level are below the diagonal.

### The dynamic of children's schoolwork motivation

**FIGURE 1:**
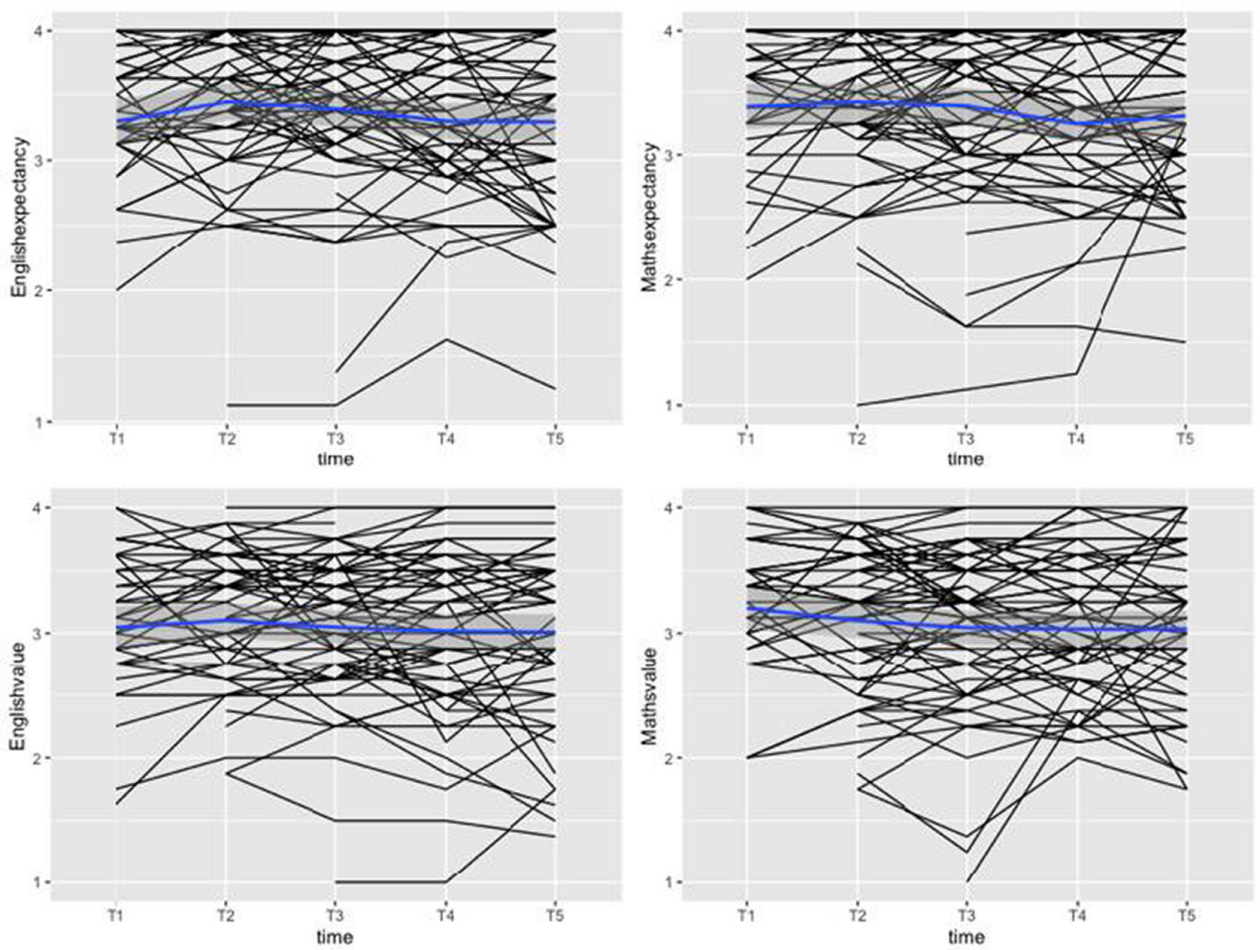
Plot of raw schoolwork motivation scores across the five weeks. *Note*: *X* axis represents timepoints during the study, *Y* axis represents motivation (expectancy and value) values [Color figure can be viewed at wileyonlinelibrary.com]

**FIGURE 2:**
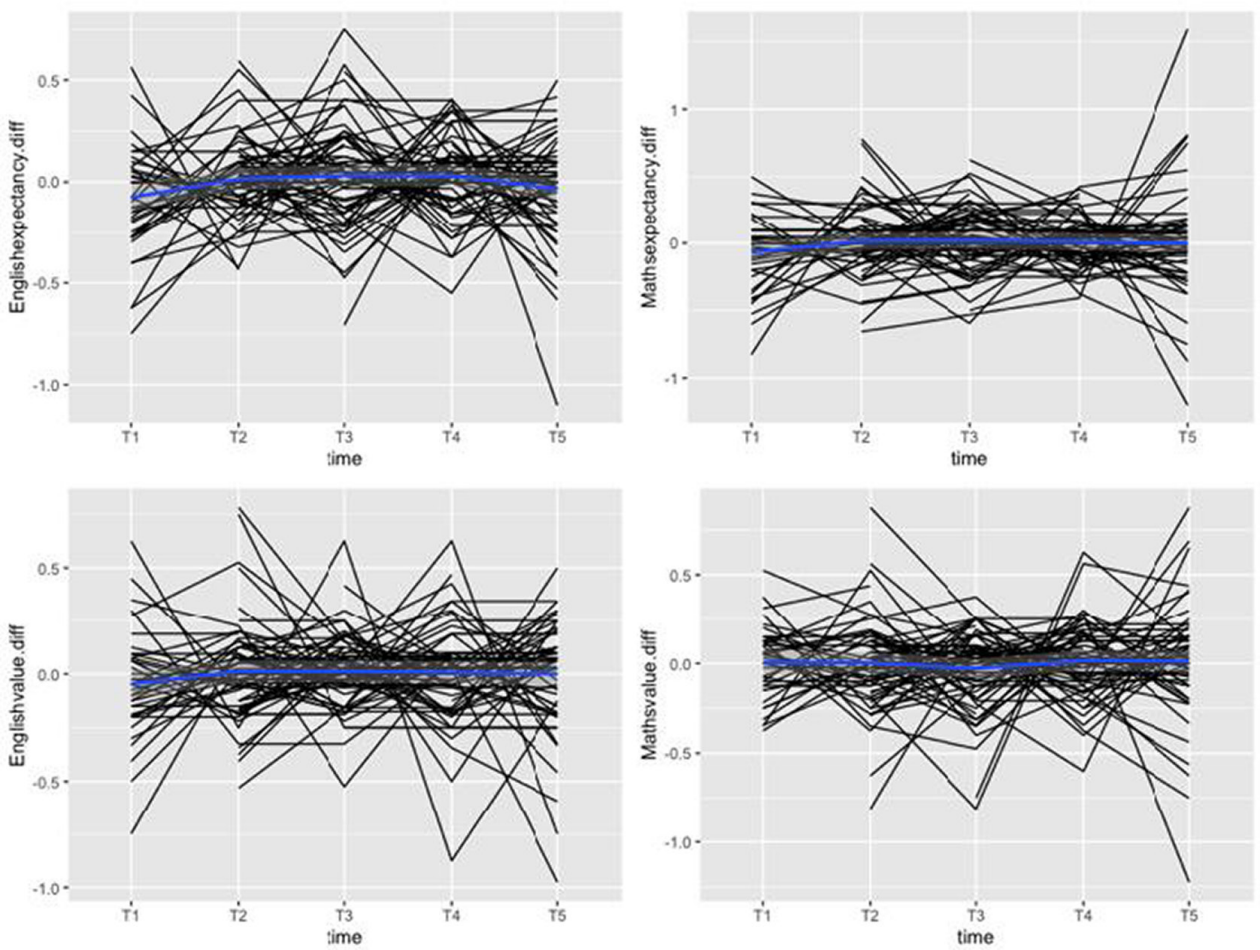
Plot of within‐person variability in schoolwork motivation. *Note*: *X* axis represents timepoints during the study, *Y* axis represents motivation (expectancy and value) within‐person variance [Color figure can be viewed at wileyonlinelibrary.com]

**FIGURE 3:**
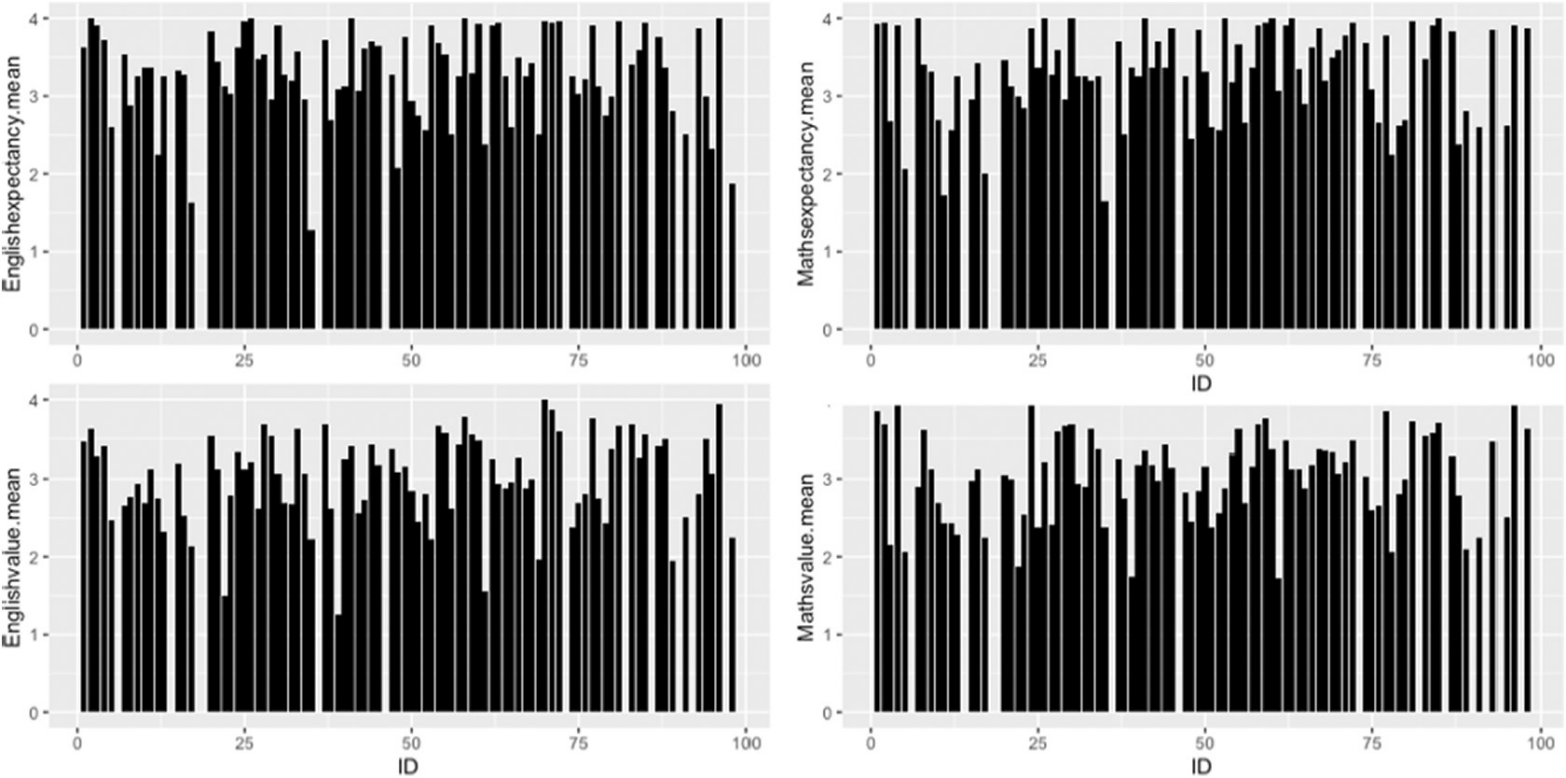
Plot of between‐person variability in schoolwork motivation. *Note*: *X* axis represents individuals, *Y* axis represents mean values of motivation (expectancy and value)

We first assessed the variability of children's schoolwork motivation over time. This variability can be examined at the level of raw data, within‐person changes (how much someone changes from wave to wave) and between‐person differences (individual differences in average levels). Figure 1 plots individual children's motivation over the five‐week study period (raw data). Figures 2 and 3 show within‐person variability and between‐person variability, respectively. Figure 1 shows that most students had high expectancy beliefs and highly valued English and maths. Few children reported low motivation at any time point. At the within‐person level (Figure 2), expectancy beliefs and schoolwork value show variation from week to week. The non‐linear line in Figure 3 shows that there is variation in expectancy beliefs and value at the between‐person level.

Intraclass correlations (ICC) were computed to decompose the variance in motivation into between‐person and within‐person components. Table S1 shows that variation in schoolwork expectancy beliefs and value is largely at the between‐person level. The ICC results for expectancy show that 67% and 64% of the variability (between‐person level variance) were attributed to the overall difference between individuals in English and maths respectively. Similarly, for the value measure, the results indicated that some variability was due to individual fluctuations over time (within‐person level), and a considerable variability was due to the overall difference between children, with 67% and 65% in English and maths, respectively.

### Relationships between children's schoolwork motivation and parental schoolwork support

To test our hypotheses, we examined the bidirectional associations between schoolwork motivation and parental schoolwork support at the between‐person and the (cross‐) lagged within‐person levels.

#### Between‐person differences

The between‐person analyses assessed whether average levels of parental support were associated with average levels of motivation (see Tables 5, 6, 7, 8). Average levels of parental support and average levels of expectancy beliefs did not significantly influence each other in English (parental English support as the predictor, *B* = 0.03; English expectancy as the predictor, *B* = −0.03) or Maths (parental maths support as predictor, *B* = 0.04; maths expectancy as the predictor, *B* = −0.03). Average levels of parental support and average levels of value beliefs did not significantly influence each other in English (parental English support as the predictor, *B* < −0.01; English value as the predictor, *B* = 0.0003) or Maths (parental maths support as the predictor, *B* = 0.02; maths value as the predictor, *B* = −0.02).

**TABLE 5:** Multilevel model of the bidirectional effect of parent support and schoolwork expectancy in English

Fixed effects	Expectancy_within_t+1_				Parent support_within_t+1_			
	Est	*SE*	*t*	*p*	Est	*SE*	*t*	*p*
Intercept	−.05	.21	−0.23	.819	.06	.23	0.25	.806
Time	−.01	.02	−0.38	.702	−.05	.02	−1.99	0.047*
Level 1 (within‐person)								
Parent support_within_t_	.02	.05	0.39	.698	−.17	.08	−2.04	.048*
Expectancy_within_t_	−.19	.10	−1.87	.063	.004	.11	0.03	.974
Level 2 (between‐person)								
Parent support_between_	.03	.04	0.92	.361	.05	.04	1.20	.232
Expectancy_between_	−.01	.04	−0.23	.820	−.03	.05	−0.59	.559
Random effects (variances)								
Level 1 (within‐person)								
Residual	.11	.33	‐	‐	.11	.33	‐	‐
Level 2 (between‐person)								
Intercept	<0.01	.002	‐	‐	.004	.07	‐	‐
Parent support	<0.01	.02	‐	‐	.10	.32	‐	‐
Expectancy	.02	.13	‐	‐	.08	.28	‐	‐

Abbreviations: Est, unstandardized estimates; *SD*, standard deviation; *SE*, standard error; t + 1, timepoint t + 1; t, timepoint t.

***p < .001, **p < .01, *p < .05.

**TABLE 6:** Multilevel model of the bidirectional effect of parent support and schoolwork expectancy in Maths

Fixed effects	Expectancy_within_t+1_				Parent support_within_t+1_			
	Est	*SE*	*t*	*p*	Est	*SE*	*t*	*p*
Intercept	−.04	.19	−0.21	.833	.16	.19	0.88	.381
Time	−.006	.02	−0.28	.782	−.07	.02	−3.34	<.001***
Level 1 (within‐person)								
Parent support_within_t_	.03	.05	0.52	.602	−.25	.05	−4.65	<.001***
Expectancy_within_t_	−.05	.09	−0.58	.559	.19	.09	2.16	.031*
Level 2 (between‐person)								
Parent support_between_	.04	.03	1.20	.232	.05	.03	1.51	.132
Expectancy_between_	−.02	.04	−0.53	.594	−.03	.04	−0.68	.500
Random effects (variances)								
Level 1 (within‐person)								
Residual	.10	.31	–	–	.10	.31	–	–
Level 2 (between‐person)								
Intercept	.000	.000	–	–	<.01	<.01	–	–
Parent support	<.01	<.01	–	–	<.01	<.01	–	–
Expectancy	<.01	<.01	–	–	<.01	<.01	–	–

Abbreviations: Est, unstandardized estimates; *SD*, standard deviation; *SE*, standard error; t + 1, timepoint t + 1; t, timepoint t.

***p < .001, **p < .01, *p < .05.

**TABLE 7:** Multilevel model of the bidirectional effect parent support and schoolwork value in English

Fixed effects	Value_within_t+1_				Parent support_within_t+1_			
	Est	*SE*	*t*	*p*	Est	*SE*	*t*	*p*
Intercept	−.03	.19	−0.13	.894	.003	.21	0.01	.989
Time	−.006	.02	−0.28	.779	−.04	.02	−1.77	.077
Level 1 (within‐person)								
Parent support_within_t_	−.003	.04	−0.07	.945	−.18	.08	−2.14	.039*
Value_within_t_	−.14	.10	−1.45	.151	.23	.16	1.39	.175
Level 2 (between‐person)								
Parent support_between_	−.0007	.04	−0.02	.984	.03	.04	0.81	.416
Value_between_	.02	.04	0.41	.684	.0003	.04	0.008	.993
Random effects (variances)								
Level 1 (within‐person)								
Residual	.09	.31	–	–	.09	.31	–	–
Level 2 (between‐person)								
Intercept	<.01	.01	–	–	.010	.08	–	–
Parent support	<.01	.05	–	–	.13	.36	–	–
Value	.02	.11	–	–	.64	.79	–	–

Abbreviations: Est, unstandardized estimates; *SD*, standard deviation; *SE*, standard error; t + 1, timepoint t + 1; t, timepoint t.

***p < .001, **p < .01, *p < .05.

**TABLE 8:** Multilevel model of the bidirectional effect parent support and schoolwork value in Maths

Fixed effects	Value_within_t+1_				Parent support_within_t+1_			
	Est	*SE*	*t*	*p*	Est	*SE*	*t*	*p*
Intercept	.03	.19	0.15	.885	.18	.18	1.00	.318
Time	.002	.02	0.08	.933	−.06	.02	−2.7	.005*
Level 1 (within‐person)								
Parent support_within_t_	−.02	.05	−0.40	.687	−.23	.05	−4.11	<0.001***
Value_within_t_	−.15	.09	−1.64	.102	−.004	.09	−0.05	.961
Level 2 (between‐person)								
Parent support_between_	.02	.03	0.60	.549	.02	.03	0.72	.472
Value_between_	−.03	.04	−0.77	.439	−.02	.04	−0.49	.625
Random effects (variances)								
Level 1 (within‐person)								
Residual	.09	.31	–	–	.09	.31	–	–
Level 2 (between‐person)								
Intercept	<.01	<.01	–	–	<.01	.01		
Parent support	<.01	<.01	–	–	<.01	.06	–	–
Value	<.01	<.01	–	–	<.01	<.01	–	–

Abbreviations: Est, unstandardized estimates; *SD*, standard deviation; *SE*, standard error; t + 1, timepoint t + 1; t, timepoint t.

***p < .001, **p < .01, *p < .05.

#### Within‐Person lagged processes

The results revealed that there was a negative effect of time on parental support (see Tables 5, 6, 7, 8) indicating that children perceived a significant reduction in parental support during five weeks (*B*'s range from −0.07 to – 0.04). The time effects on expectancy beliefs and value showed an overall decrease over time (*B*'s *range* from −0.01 to – 0.006) except for the Maths value. Results for the Maths value beliefs indicated a slightly increasing time trend. However, these changes did not differ at a statistically significant level (*B* = 0.002).

Parental schoolwork support did not predict subsequent expectancy beliefs in either Maths (*B* = 0.03, *p* = .602) or English (*B* = 0.02, *p* = .698). Parental schoolwork support also did not predict subsequent maths schoolwork value (*B* = −0.02, *p* = .687) or English schoolwork value (*B* = −0.003, *p* = .945). Thus, the results did not indicate that parental support in one week predicted children's motivational beliefs in the subsequent week.

There was some evidence that children's motivational beliefs in one week predicted perceived parental support in the subsequent week. Specifically, when children reported higher maths expectancy beliefs than usual, they reported greater perceived parental support in maths in the subsequent week (*B* = 0.19, *p* = .031). However, weekly variations in English expectancy beliefs were not significantly related to subsequent perceived parental support in English (*B* = 0.004, *p* = .974). Both value for English (*B* = 0.23, *p* = .175) and maths (*B* = −0.004, *p* = .961) did not predict subsequent levels of parent support.

Additionally, perceived parent support in one week significantly negatively predicted subsequent levels of parent support (*B's* range from −0.25 to −0.17, *p* < .05). Specifically, children tended to report lower levels of support in the subsequent weeks following a week in which they reported a high level of perceived support.

## DISCUSSION

In this study, we provided new evidence on how children's schoolwork expectancies and value vary over short time spans. First, in the novel context of home schooling during a pandemic, we found that children's motivation was consistently high during the spring lockdown period. Furthermore, we showed that the majority of variance in children's motivation (expectancy and value) was due to the difference between individuals (rather than within individuals over time). The second aim of this study was to examine the bidirectional effects between parental support and schoolwork motivation. We found that both individual differences between children and within‐person fluctuations in children's perception of parental support did not predict children's schoolwork motivation. Another finding was that higher maths expectancies predicted higher parental support for maths schoolwork at the within‐person level. Overall, these results shed new light on the association between perceived parental support and children's schoolwork motivation at the between‐person and within‐person levels.

### The variability of schoolwork motivation

When splitting variance in children's schoolwork expectancies and value, there is a substantial trait‐like proportion and a smaller within person time‐variant proportion. Most of the variance in students' expectancies and value were located at the between‐person level, which is in accordance with findings that there are large individual differences in children's motivation (Chow et al., 2012). We therefore provide evidence that it is possible to split children's motivational beliefs into trait‐like and state‐like components as has been suggested in recent theory on this topic (Eccles & Wigfield, 2020). Taking this approach in future research may improve our precision in interventions targeting motivation.

Expectancy and value beliefs were consistently high throughout the five‐week study. This may be a characteristic of the voluntary sample (i.e., more motivated children may have been willing to take part). Children lower in motivation and those without good internet access are likely less represented in our sample. However, the consistently high level of motivation in this sample points to high levels of resilience among these children. In the academic context, resilience is viewed as the student's ability to successfully adjust to adverse life events (Martin, 2013). Students who remained resilient were likely to maintain a high level of academic motivation despite a challenging and difficult environment (Oyoo et al., 2018). Furthermore, most people are resilient even when facing challenging or threatening circumstances (Chen & Bonanno, 2020). Because we do not have a representative sample of the population and relied on an opt‐in sample, we cannot assume all children showed these patterns of resilience during the lockdown. In fact, many reports suggest there were large socio‐economic discrepancies in the quality and amount of schoolwork children completed during the UK lockdown (Green, 2020).

### The bidirectional relationship between parental support and schoolwork motivation

#### The between‐person process

On the question of the association between parental support and schoolwork motivation, our study found that parental support did not predict students' expectancy or value beliefs at the between‐person level. This finding is contrary to our expectations and previous cross‐sectional and traditional longitudinal studies (with longer time lags) that have suggested that parental support seems to facilitate students' motivation (Vasquez et al., 2016). One possible explanation for the non‐significant association between parental support and schoolwork motivation might be that long hours of study with parents may cause fatigue for both the children and their parents. Perhaps parents cannot provide high‐quality support consistently, and parental support might be ineffective for schoolwork motivation. Surprisingly, there was no evidence that students' motivation has an influence on parental support. Perhaps parents modify their behaviour based on clearly observable and interpretable indicators (like performance vs. motivation). Another reason may be that these data were collected at a specific timepoint in which all schoolwork was completed at home while parents had competing demands (e.g., work).

#### The within‐person process

We hypothesized a pattern of transactional effects within families, with parents and children influencing each other over time. There was no evidence that parental support influenced children's motivation. We tested four possible effects of children's motivation on parental support, but only found support for one pathway where maths schoolwork expectancies positively predicted parental support. More specifically, when children felt they were more able to complete their maths schoolwork, they received more support from their parents in the following week. Feelings of success elicited more support from parents, possibly because children who feel able are more comfortable and confident asking for help from parents, parents find it easier to communicate and work with motivated children, or parents prefer to help their children when they succeed (Núñez et al., 2017). Such evocative effects are in accord with Sameroff's transactional model that acknowledges children's behaviour influences parental behaviour (Sameroff & Fiese, 2000). Furthermore, the main point of this model is the bidirectional effects of children and parents (Sameroff & Mackenzie, 2003). However, this finding only provides partial support of the transactional model because there was no evidence that parental support had subsequent effects on children's feelings of expectancy beliefs. In sum, children's maths expectancy beliefs appear to drive parental support behaviour in the short term (i.e., week to week), but parental support behaviour did not influence children's expectancy beliefs.

However, contrary to expectations, this study did not find significant transactional effects in English. The variation in schoolwork expectancy among students was larger in maths than in English. This difference indicates that maths schoolwork was perceived as difficult by at least some children. Thus, children might ask for more help in maths. This result may also be explained by the fact that children's expectancy and value beliefs follow domain‐specific patterns. Research has indicated that expectancy and value beliefs about different domains (maths and English) typically showed low relations (Gaspard et al., 2018). Children might show different attitudes and behaviours in English and maths.

We found no evidence of within‐person associations between schoolwork values and parental support. The lack of association may have occurred because motivational values are more stable than expectancy beliefs (Gaspard et al., 2017). Perhaps parental support influences feelings of success rather than the value placed on schoolwork. Schoolwork values may be more closely tied to parental values instead of parental support (Katz et al., 2011).

Furthermore, children perceived less parental support and had lower expectancy and value over time. Children and parents may have been overwhelmed with home learning, parents felt unable to help, or parents had limited understanding of the schoolwork material. There was also a negative association between parent support in one week and the subsequent week, which may have resulted from parents believing that their children were able to complete schoolwork independently after parents previously provided a high level of support. Thus, parents might have provided less support with schoolwork subsequently. Our findings illustrate the importance of schoolwork motivation for subsequent parental support. One practical implication is that parents should be advised to pay special attention to children who have low maths schoolwork motivation because these children are less likely to receive parental support.

### Limitations and future research

The weekly diary study design allowed us to examine the association between parental support and schoolwork motivation at between‐person and within‐person levels. Nonetheless, some limitations should be noted. First, the generalisability of these results is subject to certain limitations because our results are based on an opportunity sample from years 7 to 9. The extent to which our findings generalize to other ages is unknown. Second, this study's duration was fairly brief at only five weeks and took part in a very specific context of the first UK lockdown when children were forced to study at home rather than in school. Findings may differ under typical circumstances where children attend school in person and parental support is limited to homework outside school. It is difficult to know if the findings would generalize when children learn in school rather than at home. We suspect that parents would continue to be affected by their children even when children are in school. There is much past work suggesting that parents often help children with homework (for a review, see Wilder, 2014). Processes might have occurred at a more rapid pace and under more stressful conditions, but there is no reason to suspect that the parent–child relationship was fundamentally different during lockdown. Nonetheless, a replication during a time without lockdown is needed. In addition, lockdown may have affected these processes differently for children in different types of families. Although lockdown was difficult for most (El‐Osta et al., 2021), it was especially difficult for those from low‐income households (Waite et al., 2021). Another limitation is that we did not ask parents about their socio‐economic backgrounds, which may explain the small variance in some measures. Moreover, we only examined the relation between support and motivation on weekly basis; daily or hourly effects could be more relevant to these processes (see Malmberg & Martin, 2019). Finally, we did not separate which parent the child thought of when completing the parent support measure. Perhaps it makes a difference when it is a mother or a father providing support. Future studies are warranted to understand more fully parental support in children's schoolwork motivation.

In conclusion, at the between‐person level, there was no evidence of bidirectional effects between motivation and support; at the within‐person level, more parental support did not predict subsequent increases in motivation. However, there was some evidence supporting the presence of child‐driven effects on parental support. The present study adds to the growing body of research exploring schoolwork motivation and its bidirectional relations with parental help. This study goes beyond previous research by using an intensive weekly diary approach to assess schoolwork motivation and parental support. Taken together, the results of this study highlighted the predictive power of schoolwork expectancy on parental support. The findings suggest that children influence parents. Future research needs to incorporate an understanding of children as actors to better understand the development of academic motivation.

## AUTHOR CONTRIBUTIONS

**Yao Wu:** Conceptualization; data curation; formal analysis; investigation; methodology; project administration; software; visualization; writing – original draft; writing – review and editing. **Peter Hilpert:** Formal analysis; software; visualization; writing – review and editing. **Harriet Tenenbaum:** Conceptualization; methodology; writing – review and editing. **Terry Ng‐Knight:** Conceptualization; data curation; formal analysis; investigation; methodology; software; visualization; writing – review and editing.

## CONFLICT OF INTEREST

The authors do not have a conflict of interest.

## Supporting information


Appendix S1

## Data Availability

The data that support the findings of this study are available on request from the corresponding author. The data are not publicly available due to privacy or ethical restrictions.
