# Supplementary material for: A weekly‐diary study of students' schoolwork motivation and parental support
Source: Br J Educ Psychol. 2022 Jul 31;92(4):1667–86. doi: 10.1111/bjep.12532 (PMC9796738; doi:10.1111/bjep.12532)
Supplement: Supplementary file 1 — Appendix S1 [file BJEP-92-1667-s001.docx]

Supplementary Materials

Table S1

Between-person level variance, within-person level variance and intraclass correlation for children’s schoolwork motivation (expectancy and value) in English and Maths.

| Variable | English | | |  | Maths | | |
| --- | --- | --- | --- | --- | --- | --- | --- |
|  | Between-person Var | Within-person Var | ICC |  | Between-person Var | Within-person Var | ICC |
| Expectancy | 0.56 | 0.27 | 0.67 |  | 0.56 | 0.32 | 0.64 |
| Value | 0.54 | 0.26 | 0.67 |  | 0.52 | 0.28 | 0.65 |

*Note.* Var = variance; ICC = intraclass correlation coefficient.

Table S2

Multilevel Model of the bidirectional effect of parent-reported Parent support and Schoolwork expectancy in English.

|  | Expectancy_within_t+1_ | | | |  | Parent support_within_t+1_ | | | |
| --- | --- | --- | --- | --- | --- | --- | --- | --- | --- |
| Fixed effects | Est | SE | t | *p* |  | Est | SE | t | *p* |
| Intercept | -.01 | .18 | -0.08 | .93 |  | .08 | .17 | 0.48 | .63 |
| Time | -.001 | .02 | -0.06 | .95 |  | -.07 | .02 | -3.64 | <.001*** |
| Level 1 (within-person) |  |  |  |  |  |  |  |  |  |
| Parent support_within_t_ | .09 | .05 | 1.71 | .09 |  | -.21 | .05 | -4.21 | <.001*** |
| Expectancy_within_t_ | -.14 | .09 | -1.64 | .10 |  | -.25 | .09 | -2.92 | .003** |
| Level 2 (between-person) |  |  |  |  |  |  |  |  |  |
| Parent support_between_ | .01 | .03 | 0.31 | .75 |  | .03 | .03 | 1.02 | .31 |
| Expectancy_between_ | .003 | .04 | 0.09 | .93 |  | .03 | .04 | 0.73 | .46 |
| Residual | .08 | .29 | - | - |  | .08 | .29 | - | - |

*Note.* Est = unstandardized estimates; SE = standard error; SD = standard deviation; t = timepoint t, t+1= timepoint t+1; *** *p* < .001, ** *p* < .01, * *p* < .05.

Table S3

Multilevel Model of the bidirectional effect of parent-reported Parent support and Schoolwork expectancy in Maths.

|  | Expectancy_within_t+1_ | | | |  | Parent support_within_t+1_ | | | |
| --- | --- | --- | --- | --- | --- | --- | --- | --- | --- |
| Fixed effects | Est | SE | t | *p* |  | Est | SE | t | *p* |
| Intercept | -.09 | .22 | -0.43 | .67 |  | .25 | .21 | 1.19 | .23 |
| Time | -.01 | .02 | -0.44 | .66 |  | -.07 | .02 | -3.27 | .001** |
| Level 1 (within-person) |  |  |  |  |  |  |  |  |  |
| Parent support_within_t_ | -.03 | .06 | -0.53 | .60 |  | -.18 | .05 | -3.31 | <.001*** |
| Expectancy_within_t_ | -.04 | .10 | -0.35 | .73 |  | -.11 | .10 | -1.12 | .26 |
| Level 2 (between-person) |  |  |  |  |  |  |  |  |  |
| Parent support_between_ | .05 | .03 | 1.56 | .12 |  | -.03 | .03 | -0.99 | .32 |
| Expectancy_between_ | .02 | .05 | 0.39 | .70 |  | .01 | .05 | 0.14 | .89 |
| Residual | .12 | .35 | - | - |  | .12 | .35 | - | - |

*Note.* Est = unstandardized estimates; SE = standard error; SD = standard deviation; t = timepoint t, t+1= timepoint t+1; *** *p* < .001, ** *p* < .01, * *p* < .05.

Table S4

Multilevel Model of the bidirectional effect parent-reported Parent support and Schoolwork value in English.

|  | Value_within_t+1_ | | | |  | Parent support_within_t+1_ | | | |
| --- | --- | --- | --- | --- | --- | --- | --- | --- | --- |
| Fixed effects | Est | SE | t | *p* |  | Est | SE | t | *p* |
| Intercept | -.06 | .14 | -0.45 | .65 |  | -.03 | .14 | -0.20 | .84 |
| Time | .001 | .02 | -0.06 | .95 |  | -.07 | .02 | -3.64 | <.001*** |
| Level 1 (within-person) |  |  |  |  |  |  |  |  |  |
| Parent support_within_t_ | .08 | .05 | 1.54 | .12 |  | -.17 | .05 | -3.54 | <.001*** |
| Value_within_t_ | -.11 | .09 | -1.36 | .17 |  | .01 | .09 | 0.16 | .87 |
| Level 2 (between-person) |  |  |  |  |  |  |  |  |  |
| Parent support_between_ | <.01 | .03 | 0.07 | .95 |  | .03 | .03 | 1.13 | .26 |
| Value_between_ | .02 | .04 | 0.53 | .59 |  | .07 | .03 | 2.05 | .04* |
| Residual | .08 | .29 | - | - |  | .08 | .29 | - | - |

*Note.* Est = unstandardized estimates; SE = standard error; SD = standard deviation; t = timepoint t, t+1= timepoint t+1; *** *p* < .001, ** *p* < .01, * *p* < .05.

Table S5

Multilevel Model of the bidirectional effect parent-reported Parent support and Schoolwork value in Maths.

|  | Value_within_t+1_ | | | |  | Parent support_within_t+1_ | | | |
| --- | --- | --- | --- | --- | --- | --- | --- | --- | --- |
| Fixed effects | Est | SE | t | *p* |  | Est | SE | t | *p* |
| Intercept | .04 | .19 | 0.22 | .83 |  | .18 | .18 | 1.03 | .30 |
| Time | -.002 | .02 | -0.11 | .91 |  | -.08 | .02 | -3.56 | <.001*** |
| Level 1 (within-person) |  |  |  |  |  |  |  |  |  |
| Parent support_within_t_ | -.04 | .06 | -0.73 | .46 |  | -.19 | .05 | -3.48 | <.001*** |
| Value_within_t_ | -.15 | .10 | -1.51 | .13 |  | -.13 | .10 | -1.34 | .18 |
| Level 2 (between-person) |  |  |  |  |  |  |  |  |  |
| Parent support_between_ | .03 | .03 | 0.86 | .39 |  | -.03 | .03 | -0.97 | .33 |
| Value_between_ | -.02 | .04 | -0.46 | .64 |  | .03 | .04 | 0.68 | .49 |
| Residual | .12 | .34 | - | - |  | .12 | .34 | - | - |

*Note.* Est = unstandardized estimates; SE = standard error; SD = standard deviation; t = timepoint t, t+1= timepoint t+1; *** *p* < .001, ** *p* < .01, * *p* < .05.

# **Title:** **R-syntax (Multilevel modelling)**

#**List the syntax for one model. Replace the variable name for other three models.**

# STRUCTURE

# 1) Data Preparation

# 2) Analyse Models

# --------------------------------------

# 1) Data Preparation

# --------------------------------------

# remove all objects from your workspace

# rm(list=ls())

# Install the package

install.packages(“lme4”)

install.packages(“tidyverse”)

install.packages(“parameters”)

# Activate the package

library((“lme4”)

library((“tidyverse”)

library((“parameters”)

# Set your working directory. This has to be adjusted to your file source location.

setwd(“~/Desktop/”)

# Load the data files from your working directory

df <- read.csv("Desktop/lag.file.csv", header = TRUE, na.strings = c(NA))

# Prepare data: expectancy and parental support in English

# Select some variables out of the data set (model 1)

df.Eexpectancy <- select(df, ID, time, Englishexpectancy.mean, Englishexpectancy.diff,

Englishparentsupport.mean, Englishparentsupport.diff,

Englishparentsupport.diff.lead1)

names(df.Eexpectancy)[7] <- 'new.name' # rename this variable

# Select some variables out of the data set (model 2)

df.parentalsupport <- select(df, ID, time, Englishexpectancy.mean, Englishexpectancy.diff,

Englishparentsupport.mean, Englishparentsupport.diff,

Englishexpectancy.diff.lead1)

names(df.parentalsupport)[7] <- 'new.name' # rename this variable

# add dummy code

df.Eexpectancy$var <- NULL # create a new variable

df.Eexpectancy$var = 0 # add the value 0

df.parentalsupport$var <- NULL # create a new variable

df.parentalsupport$var = 1 # add the value 1

# combine two datasets into one data set

df.new1 <- rbind(df.Eexpectancy, df.parentalsupport)

# --------------------------------------

# 2) Data Analysis

# --------------------------------------

# Analyse model 1. Include dummy code (var) for computing both DVs at the same time

mod1 <- lmer(new.name ~ -1 +

var/(time + Englishparentsupport.mean +

Englishparentsupport.diff +

Englishexpectancy.mean +

Englishexpectancy.diff) +

(-1 + var/(Englishparentsupport.diff + Englishexpectancy.diff)| ID),

data = df.new1, verbose = TRUE, REML = TRUE)

summary(mod1)

parameters::p_value(mod1)
